# Draft Genome Sequence of Actinomyces neuii UMB1295, Isolated from the Female Urinary Tract

**DOI:** 10.1128/MRA.00402-20

**Published:** 2020-06-04

**Authors:** Tijana Markovic, Taylor Miller-Ensminger, Adelina Voukadinova, Alan J. Wolfe, Catherine Putonti

**Affiliations:** a Neuroscience Program, Loyola University Chicago, Chicago, Illinois, USA; b Department of Biology, Loyola University Chicago, Chicago, Illinois, USA; c Bioinformatics Program, Loyola University Chicago, Chicago, Illinois, USA; d Department of Microbiology and Immunology, Stritch School of Medicine, Loyola University Chicago, Maywood, Illinois, USA; e Department of Computer Science, Loyola University Chicago, Chicago, Illinois, USA; University of Rochester School of Medicine and Dentistry

## Abstract

Actinomyces neuii is an opportunistic pathogen. Within the urogenital tract, it has been associated with bacterial vaginosis and overactive bladder symptoms. Here, we investigate a draft genome sequence of A. neuii UMB1295, which was isolated from a catheterized urine sample from a woman with a urinary tract infection.

## ANNOUNCEMENT

Actinomyces neuii is a Gram-positive opportunistic bacterial pathogen. While A. neuii infections are generally regarded as rare, A. neuii can cause infections in multiple different organs (see reviews in references 1 and 2). A. neuii is frequently found within the female urogenital tract. Even though it has been found within the vaginal microbiota of healthy women (3), it is associated with bacterial vaginosis, and recent evidence suggests that it may enhance Gardnerella vaginalis virulence (4). Within the urinary tract, it is most frequently found in women with overactive bladder symptoms (5, 6). A. neuii UMB1295 was isolated from a catheterized urine sample obtained from a woman with a urinary tract infection (UTI) who was seeking clinical care at the Loyola University Medical Center Female Pelvic Medicine and Reconstructive Surgery Center (Maywood, IL, USA) as part of a prior institutional review board (IRB)-approved study (7) (Loyola University Chicago IRB approval no. 206469).

A. neuii UMB1295 was isolated using the expanded quantitative urinary culture (EQUC) protocol (5). The genus and species designations were determined by matrix-assisted laser desorption ionization–time of flight (MALDI-TOF) mass spectrometry (5). The A. neuii strain was streaked onto a Columbia nalidixic acid (CNA) agar plate using the quadrant streaking method and was incubated at 35°C in 5% CO_2_ for 48 h. LB broth was then inoculated with a single colony from this plate and grown under the same conditions. DNA was extracted using the DNeasy blood and tissue kit, following the Gram-positive protocol with the following exceptions: we used 230 μl of lysis buffer (180 μl of 20 mM Tris-Cl, 2 mM sodium EDTA, and 1.2% Triton X-100 and 50 μl of lysozyme). The DNA was quantified using a Qubit fluorometer and sent to the Microbial Genome Sequencing Center (MiGS) at the University of Pittsburgh for sequencing. There, it was first enzymatically fragmented with an Illumina tagmentation enzyme, and indices were attached using PCR. The library was sequenced using the Illumina NextSeq 550 platform, producing 1,380,900 pairs of 150-bp reads. The reads were trimmed using Sickle v1.33 (https://github.com/najoshi/sickle) and assembled using SPAdes v3.13.0 with the only-assembler option for k values of 55, 77, 99, and 127 (8). The genome coverage was calculated using BBMap v38.47 (https://sourceforge.net/projects/bbmap). The NCBI Prokaryotic Genome Annotation Pipeline (PGAP) v4.11 (9) was used to annotate the publicly available genome assembly. PATRIC v3.6.3 was also used to annotate the genome sequence (10). The default parameters were used for each software tool.

The A. neuii UMB1295 draft genome is 2,174,960 bp long in 21 contigs with a GC content of 56.59%, genome coverage of 159×, and an *N*_50_ score of 193,074 bp. PGAP identified 1,968 coding sequences, with 1,953 coding for proteins, 48 tRNAs, 3 complete 5S rRNAs, 1 complete 16S rRNA, and 1 complete 23S rRNA. One CRISPR array with 32 spacer sequences was identified by CRISPRCasFinder (11). While PATRIC identified 24 genes associated with antibiotic resistance, further investigation of the genome assembly by ResFinder v3.2 (12) did not reveal any predicted resistances.

The A. neuii UMB1295 genome is just the sixth genome for this species and the third from the urinary microbiome (13). Further analysis of this genome and comparison between available genomes will greatly improve our understanding of this species, including its role in the female urogenital tract.

### Data availability.

This whole-genome shotgun project has been deposited in GenBank under the accession no. JAAUWI000000000. The version described in this paper is the first version, JAAUWI010000000. The raw reads have been deposited in SRA under the accession no. SRR11441016.
